# Ovarian Seromucinous Borderline Tumor and Clear Cell Carcinoma: An Unusual Combination

**DOI:** 10.1155/2015/690891

**Published:** 2015-05-13

**Authors:** Eriko Nakamura, Yuichiro Sato, Sayaka Moriguchi, Atsushi Yamashita, Takashi Higo, Yujiro Asada

**Affiliations:** ^1^Department of Pathology, University of Miyazaki, 5200 Kihara, Kiyotake, Miyazaki 889-1692, Japan; ^2^Department of Diagnostic Pathology, Miyazaki University Hospital, University of Miyazaki, 5200 Kihara, Kiyotake, Miyazaki 889-1692, Japan; ^3^Department of Gynecology and Obstetrics, Koga Hospital, 1749-1 Sudaki, Ikeuchi, Miyazaki 880-0041, Japan

## Abstract

Ovarian seromucinous borderline tumors (SMBTs) are rare. They architecturally resemble serous borderline tumors but are much more frequently associated with endometriosis. The coexistence of other tumors with seromucinous tumors is also extremely rare. Here, we report an unusual combination of bilateral ovarian SMBT and clear cell carcinoma associated with polypoid endometriosis of the colon, in a 62-year-old woman. There was no transitional lesion between the two tumors. Immunohistochemistry showed different staining patterns in tumor components. Seromucinous tumor cells were positive for estrogen receptor (ER) and progesterone receptor (PgR) but negative for Napsin A, p504S, and HNF1B. Clear cell tumor cells were positive for Napsin A and p504S and focally positive for HNF1B but negative for ER and PgR. Loss of ARID1A expression was not observed in SMBTs, clear cell tumors, or endometriosis. These findings suggest that these tumors arose from separate endometriosis foci and collided within the same ovary. To the best of our knowledge, this is the first case of this unusual combination of ovarian seromucinous tumor and clear cell carcinoma to be reported in the English literature.

## 1. Introduction

Seromucinous borderline tumors (SMBTs) are characterized by papillary architecture reminiscent of serous tumors but composed of mucinous epithelium similar to that of the endocervix. These tumors are associated with endometriosis [1–3]. SMBTs have been considered a subset of mucinous tumors and account for 15% of all mucinous borderline tumors [1]. A few studies have reported tumors coexisting with SMBTs, such as endometrioid adenocarcinoma [4] and squamous cell carcinoma [5]. Here, we describe the first case, to our knowledge, of an unusual combination of ovarian SMBT and clear cell carcinoma. Additionally, we found typical endometriosis and an atypical endometrial nodular lesion in the colon in this patient. We suggest that these ovarian tumors and colonic lesions were also associated with separate endometriotic foci.

## 2. Case Report

A 62-year-old woman visited the hospital with a simple hepatic cyst and hepatic dysfunction. An abdominal computed tomography scan revealed bilateral ovarian tumors and uterine leiomyoma. The patient underwent a hysterectomy, bilateral salpingo-oophorectomy, and omentectomy. A nodular lesion was found at the serosa of the sigmoid colon during the surgery, and a partial sigmoid colon resection was performed. An intraoperative diagnosis with frozen section was not performed. However, this case was diagnosed as stage Ib disease (FIGO staging). The patient received chemotherapy with paclitaxel and carboplatin. At four years after surgery, no local recurrence or metastasis has been found clinically.

**Figure 1:**
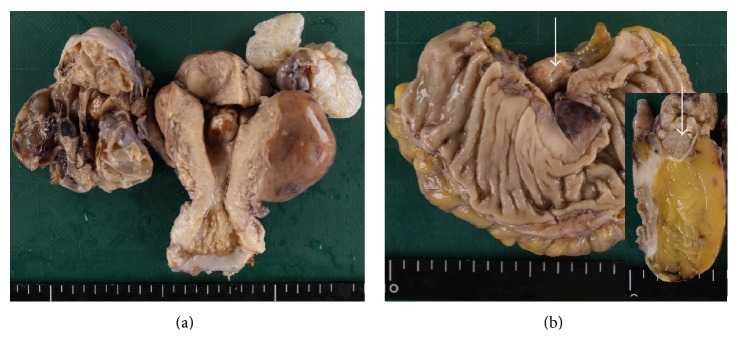
Macroscopic findings for ovaries (a) and sigmoid colon (b). (a) The ovaries exhibited multiple cysts that contained mucoid material but were not hemorrhagic or necrotic (b). The nodular colonic lesion was located in the serosa (arrow); its cut surface was spongy (inset image).

The right ovarian tumor was 7 cm at its greatest diameter; its cut surface was characterized by multiple cysts with mucinous, faintly brown fluid (Figure 1(a)). The left ovarian tumor was 4 cm; its cut surface was spongy with multiple cysts. A 2 cm sigmoid tumor with a spongy cut surface was located in the colon serosa (Figure 1(b)).

**Figure 2:**
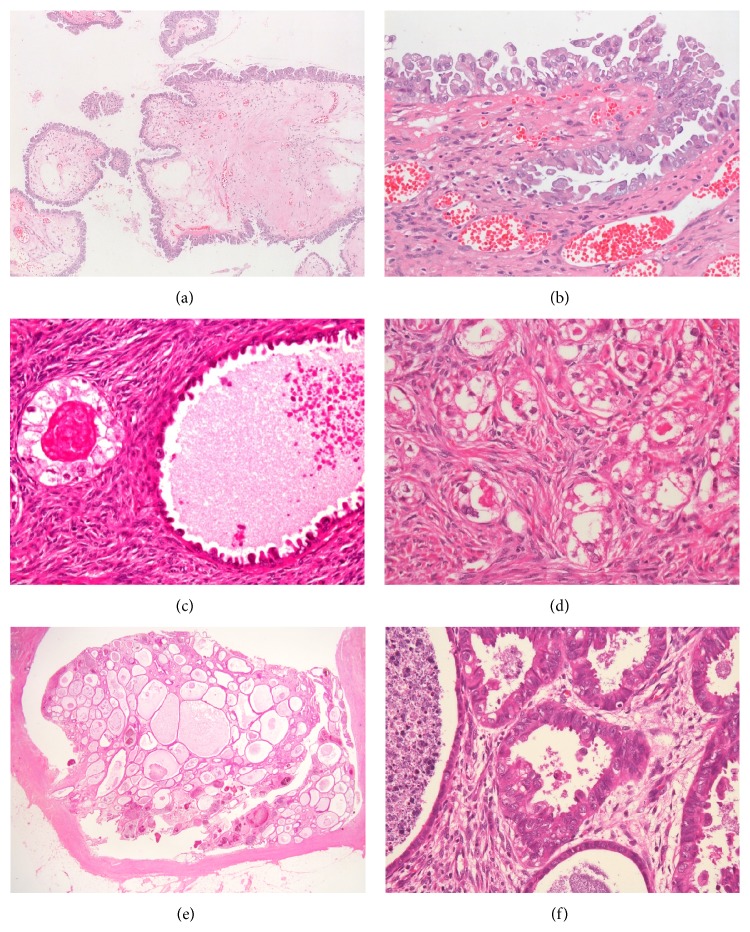
Microscopic findings for ovaries (a–d) and the sigmoid colon (e, f). (a, b) The papillary tumors of both ovaries were lined by tall columnar mucinous and ciliated serous cells. (c) The clear cell tumors had clear cytoplasm and a hobnail-shaped appearance. (d) Small clusters of clear cell tumor cells with nuclear atypia were surrounded by desmoplastic stroma. (e) The nodular colonic lesion was composed of dilated endometrioid glands. (f) Focal atypical glands with some papillary structure and nuclei.

Microscopically, multiple cystic lesions or exophytic growth with low papillary lesions was identified in the ovaries (Figure 2(a)). Many of the cysts and the low papillary growth lesions were lined by tall columnar mucinous or cuboidal ciliated serous cells (Figure 2(b)). Eosinophilic cytoplasm and focal neutrophilic infiltration were present. Nuclear stratification was noted, but no invasive growth was apparent in these cystic tumors. Clear cell tumors were also found in both ovaries. These tumor cells had clear cytoplasm and a hobnail-shaped appearance (Figure 2(c)). The tumors showed glandular proliferation or small nests with invasive growth (Figure 2(d)). No apparent transitional lesions were noted between the different tumor components. Typical endometriosis lesions were present in both ovaries. The colonic tumor exhibited crowded endometrial glands (Figure 2(e)) as well as variation in the glandular architecture and cytology (Figure 2(f)). Typical endometriosis was also noted in the colon wall.

**Figure 3:**
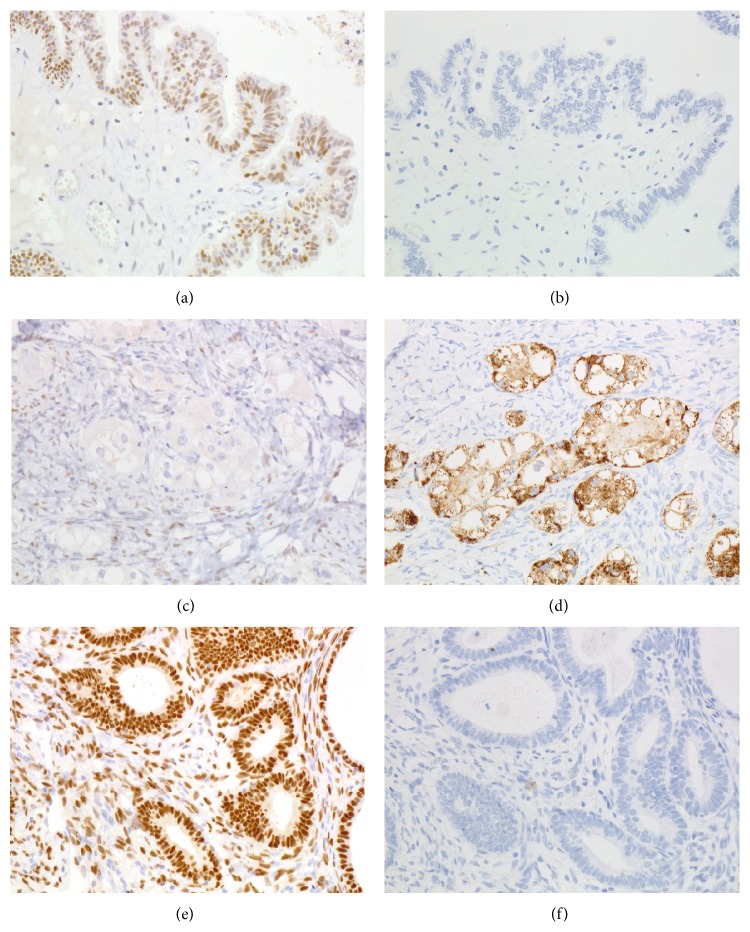
Immunohistochemical study for (a, b) seromucinous tumor, (c, d) clear cell carcinoma, and (e, f) polypoid endometriosis. Estrogen receptor: (a), (c), and (e). Napsin A: (b), (d), and (f). Seromucinous tumor cells and endometriosis cells are positive for estrogen receptor (a, e) but negative for Napsin A (b, f). Clear cell carcinoma cells are positive for Napsin A (d) but negative for estrogen receptor (c).

Immunohistochemical studies demonstrated ovarian SMBT cells, and sigmoid colon glandular cells were positive for estrogen receptor (ER) and progesterone receptor (PgR) but negative for Napsin A, p53, and HNF1B (Figures 3(a), 3(b), 3(e), and 3(f)). The clear cell tumor cells were positive for Napsin A and weakly positive for HNF1B and p53, but negative for ER and PgR (Figures 3(c) and 3(d)). The MIB-1 ratio was 18% for the clear cell carcinoma, 8% for the SMBT cells, and 5% for the sigmoid colon glandular cells. We did not observe loss of ARID1A expression in SMBT, clear cell tumor, or endometriotic tissue.

## 3. Discussion

SMBTs are grossly, microscopically, and immunohistochemically distinct from gastrointestinal-type mucinous borderline tumors [1–3, 6]. SMBTs are much less common, smaller, and more frequently bilateral; they architecturally resemble serous borderline tumors and are much more frequently associated with endometriosis. The microscopic findings of the present case were consistent with SMBT. These epithelial cells were also positive for CK7, ER, and PgR, but negative for CK20 and CDX2 [6].

The coexistence of ovarian SMBT with other carcinomas is rare. Dubé et al. [4] reported a case of SMBT with endometrioid/clear cell adenofibroma. D'Angelo et al. [5] reported a case of squamous cell carcinoma that arose from a SMBT in the same ovary (Table 1). In the present case, we identified clear cell carcinoma and SMBT within the same ovaries (on both sides). To our knowledge, this is the first report of coexisting ovarian SMBT and clear cell carcinoma in the English literature.

The association of clear cell carcinoma with mucinous lesions has rarely been described [7, 8]. In a previous case report, Wani and Notohara [8] concluded that an identified ovarian clear cell carcinoma had arisen from a mucinous cystadenoma because this case exhibited a unilocular cyst without a solid mass and demonstrated a histologic continuum between the clear and mucinous components. The present case exhibited no transitional lesion between the clear and SMBT components. Furthermore, we identified different immunophenotypes between the clear and SMBT components. Ovarian tumors that arise in the presence of endometriosis are usually endometrioid—often, clear cell carcinoma—but are rarely serous, mucinous, or seromucinous. We suggest that clear cell carcinoma and seromucinous tumors may arise from separate foci of endometriosis and collide within the same ovary.

Pathogenesis for endometrium-related tumors is not established.* ARID1A*, a recently identified tumor suppressor, frequently becomes muted and loses its expression in endometrium-related carcinomas, including ovarian clear cell and ovarian endometrioid carcinomas [9]. Wu et al. [10] reported loss of ARID1A expression in a third of seromucinous tumors. In this case, the SMBT, clear cell tumor, and endometriosis samples showed no loss of expression and/or mutation of* ARID1A*. Our results cannot support the* ARID1A*-related pathway.

Napsin A is a known diagnostic marker for lung adenocarcinoma and is expressed in some thyroid, renal, and endometrial cancers [11, 12]. Recently, Skirnisdottir et al. [13] described Napsin A as a useful marker for diagnosis of ovarian clear cell adenocarcinoma. In the present case, clear cell adenocarcinoma cells were positive for Napsin A, but no Napsin A expression was observed in seromucinous tumor and endometrioid polypoid lesion. Therefore, the findings of our case support that Napsin A is a sensitive and specific marker of clear cell carcinoma in ovaries.

Ovarian SMBTs lacking stromal invasion were initially described by Rutgers and Scully [1], who proposed the terms Müllerian mucinous or endocervical papillary cystadenoma of borderline malignancy. Several authors have reported intraepithelial or invasive carcinoma of these seromucinous tumors. Although Shappell et al. [2] reported the deaths of two patients from stage III invasive carcinomas, nearly all patients with these tumors were alive. Our patient, with stage Ib ovarian mixed SMBT and clear cell carcinoma, is alive at 4 years postoperatively, and no local recurrence or metastasis has been noted clinically.

The term polypoid endometriosis was first used by Mostoufizadeh and Scully [14] to describe an uncommon and distinctive variant of endometriosis with histologic features simulating those of an endometrial polyp. This variant of endometriosis occurs frequently in postmenopausal women; pelvic lesions are typically seen in the colon, ovary, uterine serosa, and cervical or vaginal mucosa. Parker et al. [15] reported 24 patients with polypoid endometriosis, most of whom were referred because of difficulty in making the diagnosis. The polypoid colonic lesion observed in our case was a well-demarcated nodular lesion composed of endometrial glands and stroma. Focal architectural and cellular atypia of the glandular epithelium was seen, but the lesion was distinguishable from a well-differentiated adenocarcinoma or metastatic carcinoma because it lacked an invasive pattern, cytologically malignant epithelium, and vessel permeation.

In summary, we have described the first case of an ovarian mixed tumor consisting of clear cell carcinoma and SMBT, which occurred together with polypoid endometriosis of the colon, and we have shown that these lesions can be associated with endometriosis.

## Figures and Tables

**Table 1:** Reported cases and the present case of SMBT coexisting with other tumors.

Reference	Age	Stage	Size (cm)	Endometriosis	Bilateral ovaries	Associated disease	Follow-up (M)
Dubé et al. [4]	51	Ic	10	+	−	Endometrioid/clear cell adenofibroma in ipsilateral ovary	NED (6)

D'Angelo et al. [5]	58	Ia	13	−	−	Squamous cell carcinoma	NED (4)

Present case	62	Ib	R.: 7 cm/L.: 4 cm	+	+	Clear cell carcinoma in both ovaries	NED (48)

M: months; NED: no evidence of disease.
